# Biomechanical Aspects of Closing Approaches in Postcarotid Endarterectomy

**DOI:** 10.1155/2018/4517652

**Published:** 2018-10-28

**Authors:** Idit Avrahami, Dafna Raz, Oranit Bash

**Affiliations:** Department of Mechanical Engineering and Mechatronics, Ariel University, Ariel, Israel

## Abstract

The carotid bifurcation tends to develop atherosclerotic stenoses which might interfere with cerebral blood supply. In cases of arterial blockage, the common clinical solution is to remove the plaque via carotid endarterectomy (CEA) surgery. Artery closure after surgery using primary closures along the cutting edge might lead to artery narrowing and restrict blood flow. An alternative approach is patch angioplasty which takes longer time and leads to more during-surgery complications. The present study uses numerical methods with fluid-structure interaction (FSI) to explore and compare the two solutions in terms of hemodynamics and stress and strain fields developed in the artery wall.

## 1. Introduction

The carotid bifurcation tends to develop atherosclerotic stenosis, which might interfere with cerebral blood supply and can cause coma, hemodynamic disturbance, stroke, and even death. The common clinical solution is to remove the plaque via carotid endarterectomy (CEA) surgery [1], with approximately 100,000 CEAs performed in the United States each year [2–4]. There are few approaches for postsurgery closure [5], and the preferred closure technique still remains an issue of debate [6, 7].

The CEA procedure often associated with intimal hyperplasia [8] or progression of atherosclerosis in the zone of arterial reconstructions [9], attributed to the inflammatory process due to foreign materials in vascular reconstruction [10].

A regular suture (primary closure) is the simplest way to close an arteriotomy, which leads to smaller artery diameter and increased stiffness of the structure. Therefore, routine use of patch graft has been advocated to reduce restenosis, stroke, and death [11, 12]. Patch angioplasty reduces the risk of immediate postoperative complications, results in a larger carotid artery diameter, and significantly lowers vessel restenosis and occlusion rates [13], especially in women [14].

However, the procedure of patch suturing takes longer and thus might increase the risk of stroke and death during surgery. Moreover, a concern is raised about the thrombogenic nature of the conventionally used patches and their protective effect, particularly from late restenosis [15]. Patch angioplasty has also been associated with specific complications, such as patch rupture or expansion and increased risk of infection [16]. In addition, carotid patch was shown to promote irregular neointimal lining with prominent proliferative activity [17].

According to a statistical study by Mannheim et al. [18], a 2-year restenosis-free rate is 97.6% for patch angioplasty vs. 90.9% for primary closure. Nevertheless, overall mortality or morbidity is similar for all closure procedures [13, 19, 20]. Therefore, the use of patch grafts today is targeted selectively only to patients who have very small (<4 mm) or highly constricted and tortuous vessels [7, 20].

There are several types of patches such as prosthetic patches (woven Dacron or PTFE), venous patches, and biomaterial patches (bovine pericardium) [6, 21]. Vein patch is considered the preferred one, thanks to its high compliance and biocompatibility [14]; its main disadvantage is the need for an additional incision to obtain it and occasional deterioration with aneurysmal dilatation and rupture [16]. The advantages of prosthetic patches include immediate availability, avoidance of additional incision, and preservation of vein for future use in other cardiovascular operations. The main shortcomings of the prosthetic patches are higher thrombogenicity, increased risk for infection [22], and higher risks for infections [6]. Bovine pericardium patches recently proved high durability and better long-term survival rates, compared to the other patches [23]. Overall, despite the preference of using biological patches, the clinical results with most available synthetic patches are currently similar enough to prevent clear recommendation of any particular one [6].

Some studies used computational fluid dynamics (CFD) to investigate the blood flow regime in the post-CEA region in patient-specific geometries. Harrison et al. [24] showed that incorporation of a patch indeed increases the artery diameter, but it results with larger areas of low-wall shear stress (WSS) and high-oscillatory shear index (OSI) at the bifurcation, and therefore, its benefit is questionable. Similarly, Guerciotti et al. [25] and Domanin et al. [26] also analyzed WSS, vorticity, time-averaged OSI, and relative residence time (RRT). According to all these studies, cases with primary suture resulted with better hemodynamic parameters and smaller areas of disturbed flow in comparison with patch graft cases. Especially, OSI and RRT values were generally higher in patch graft cases with respect to primary closure, especially for high carotids or when the arteriotomy is mainly at the bulb region.

Although these studies discussed the effect of the closing approach on blood flow hemodynamics to support the clinical decision and to provide a hemodynamic insight into the patch complications, they neglected the biomechanical effect on the arterial tissue and the artificial graft. Excessive stress and cyclic strains are correlated to risk of patch rapture, aneurysms, wall injury, restenosis, irregular neointimal lining [27]. Kamenskiy et al. [22] used fluid-structure interaction (FSI) numerical simulations to explore tissue strain and stress in addition to WSS parameters for models with different patch types, widths, and location. In their study, they showed that narrow patches are superior to wide patches, and anterior arteriotomy are superior to lateral arteriotomy.

As far as we know, no study examined the biomechanical aspects of patch angioplasty in comparison to primary suture. In this study, we use FSI numerical models in order to examine the hemodynamics and biomechanical aspects of the patch procedure in comparison with primary suture.

## 2. Methods

### 2.1. Cases Studied and Models Geometry

The research study used FSI numerical simulations to explore blood flows and wall dynamics in five different models of the carotid artery region. The examined cases are listed in Table 1.

**Figure 1:**
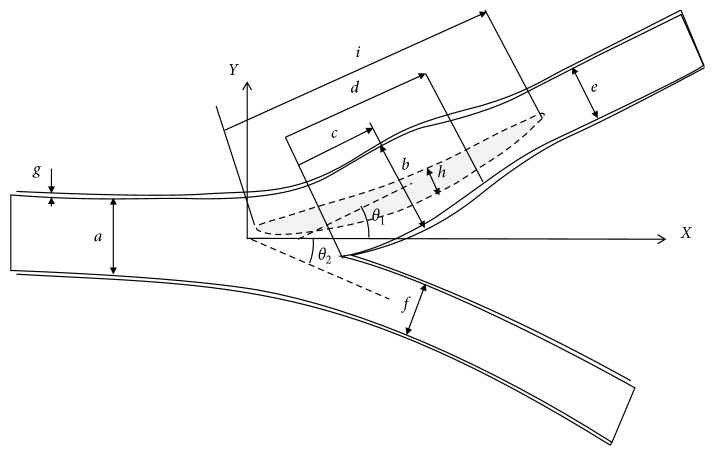
Geometry of the artery bifurcation.

**Figure 2:**
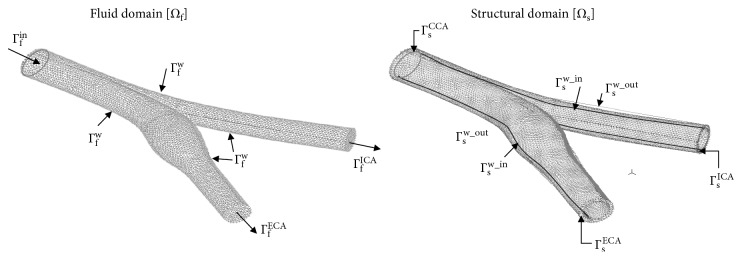
The fluid (*Ω*_f_) and Structural (*Ω*_s_) domains and the corresponding boundary conditions (specified in Equations (1)–(3)).

The geometric specifications of healthy and postsurgery bifurcation models were based on data specified by Tada and Tarbell [28] and Halak et al. [29]. The geometries were idealized to represent a typical model within the framework of the anatomy variance. Geometry dimensions are shown in Figure 1 and detailed in Table 2. The fluid and the structural domains of the model are shown in Figure 2.

### 2.2. Mathematical Model

Blood was assumed homogenous and Newtonian fluid with viscosity of *µ* = 0.0035 gr/cm·s and density of *ρ* = 1.05 g/cm^3^. The flow was assumed laminar, and the arterial wall was assumed linearly elastic with an elasticity of *E* = 5 × 10^6^ dyn/cm^2^ and Poisson's ratio of *ν* = 0.499 [30]. Small displacement/small-strain formulation was assumed. Patches and suture parameters are listed in Table 3.

The flow and pressure fields in the fluid domain (*Ω*_f_) were calculated by solving the governing equations for the fluid domain for laminar, Newtonian, and incompressible flow in a nongravity field:

(1) $$\begin{array}{c} \left.\begin{array}{c} \nabla \cdot U_{\text{f}}=0,\\ \rho_{\text{f}}\left(\frac{\partial U_{\text{f}}}{\partial t}+U_{\text{f}}\cdot \nabla U_{\text{f}}\right)=-\nabla P+\mu \cdot \nabla^{2}U_{\text{f}}, \end{array}\right\}\;\text{in}\;\;\Omega_{\text{f}}, \end{array}$$

where *P* is the static pressure, **U**_f_ is the velocity vector, *t* is the time, *ρ*_f_ is the fluid density, and *μ* is the dynamic viscosity.

The governing equation for the solid domain (*Ω*_s_) is the Lagrangian momentum conservation equation:

(2) $$\begin{array}{c} \left.\rho_{\text{s}}\frac{\partial^{2}d_{\text{s}}}{\partial t^{2}}-\nabla \sigma_{\text{s}}=f\right\}\;\text{in}\;\;\Omega_{\text{s}}, \end{array}$$

where **σ**_s_ is the Cauchy stress tensor, **d**_s_ is the vector of structure displacement, *ρ*_s_ is the wall density, and **f** represents the body force applied on the structure.

**Figure 3:**
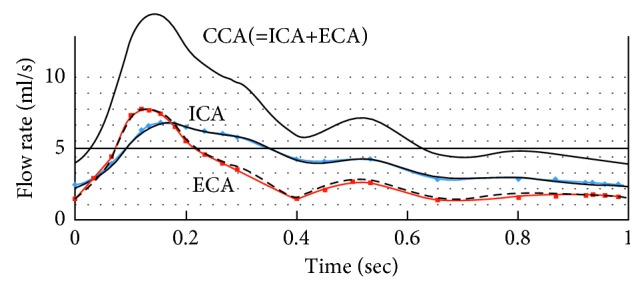
Time-dependent flow at the three arteries outlets (CCA, ICA, and ECA) [30].

**Figure 4:**
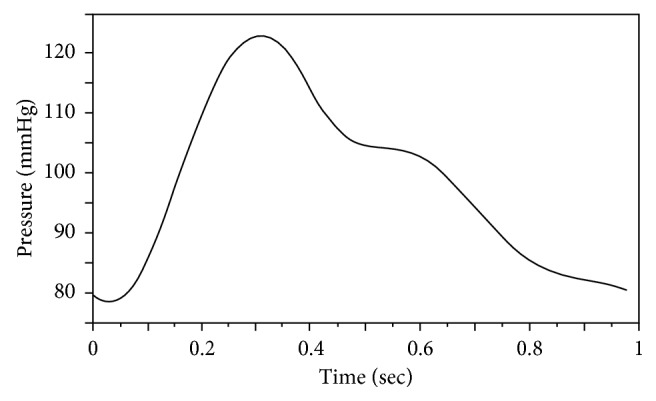
Imposed arterial pressure as a function of time [30].

We used artificial boundary conditions (BC) [22, 31] for the fluid domain as follows. Prescribed velocity conditions ∫(**U** · **n**) *dA*=*Q*(*t*) were imposed at the ICA and the ECA outlets (marked as Γ_f_^ICA^ and Γ_f_^ECA^ in Figure 2), reflecting a typical physiological waveform [30] of a normal healthy human with a heart rate of 60 bpm, as shown in Figure 3. At the CCA inlet (Γ_f_^in^), stress free conditions were employed, thus the CCA flow was achieved from mass conservation. In addition, a typical physiological time-dependent pressure was imposed, as shown in Figure 4, based on a systolic/diastolic pressure of 120/80 mmHg:

(3) $$\begin{array}{c} \left.n\cdot \tau_{\text{f}}=0,\;\;P=P\left(t\right)\right\}\;\text{on}\;\;\Gamma_{\text{f}}^{\text{in}}, \end{array}$$

where **n** · **τ**_f_ are the normal stresses to the surface. BC on the structural domains were fixed (${\dot{d}}_{\text{s}}=0$) at the edges Γ_s_^CCA^, Γ_s_^ECA^, and Γ_s_^ICA^, and stress-free conditions ($\partial {\dot{d}}_{\text{s}}/\partial n=0$) were set at the outer faces Γ_s_^w_out^.

The BC at the FSI interfaces (Γ_f_^w^ and Γ_s_^w_in^) states that (i) displacements of the fluid and solid domain must be compatible, (ii) tractions at these boundaries must be at equilibrium, and (iii) fluid obeys the no-slip\no-penetration conditions. These conditions are given in the following equations:

(4) $$\begin{array}{c} \left.\begin{array}{c} d_{\text{f}}=d_{\text{s}},\;\;U_{\text{f}}={\dot{d}}_{\text{s}},\;\;\\ n\cdot \tau_{\text{f}}=n\cdot \sigma_{\text{s}}, \end{array}\right\}\;\text{on}\;\;\Gamma_{f}^{\text{w}}\;\;\mathrm{and}\;\;\Gamma_{\text{s}}^{\text{w\_in}}, \end{array}$$

where **σ**_s_, **τ**_f_, **d**_s_, and **d**_f_ are the wall structure, fluid stress tensors, and wall displacement, respectively.

### 2.3. Numerical Model

The simulations used the commercial package ADINA (ADINA R&D, Inc., v. 9.0.0) to solve numerically the governing differential equations (Equations (1) and (2)) using the finite elements method (FEM).

The fluid domain (*Ω*_f_) was meshed using 3D 1^st^ order tetrahedral elements, and the structural domain was meshed using triangular 3D 2^nd^ order tetrahedral elements. Several mesh and time-step independence tests were conducted, where the simulations results obtained from several numerical models with different mesh and time steps were compared (Appendix). Based on these tests, the numerical model selected was with ∼100,000 fluid elements and ∼20,000 structural elements. Each cardiac cycle consisted of 28 time steps of 0.035 sec, and the automatic-time-stepping procedure was used to subdivide the load-step increment when necessary. Three cardiac cycles were computed to obtain results independent of the initial conditions. The results of the third calculated cycle were fully periodic. Convergence is achieved when all mass, velocity component, and energy changes, from iteration to iteration, achieved are less than 10^−5^ root-mean-square error (RMSE).

**Figure 5:**
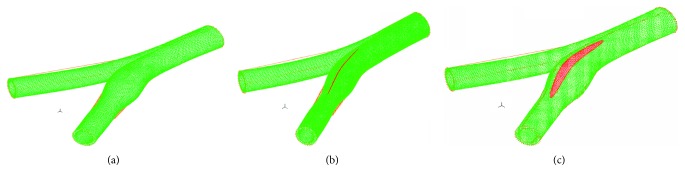
Mesh models of the healthy artery (a), suture (b), and patch (c).

The three mesh models (of the healthy, suture, and patch) are shown in Figure 5. The suture or the patch elements (marked in red in the figure) were fully connected to the arterial elements (marked in green). In the suture model, 817 elements were defined as suture, and in the patch models, 5790 elements were defined as patches. The suture or the patch elements were defined as fully connected to the arterial elements.

The ADINA iterative solver was used for the FSI coupling [32]. The fluid and the structure solvers were solved iteratively until convergence was reached or until it reached 150 iterations. The FSI algorithm included models of linear wall displacements and strains. In order to control the moving mesh under deformations of the flow domains, the arbitrary Lagrangian-Eulerian (ALE) approach was defined on geometric entities. The ALE approach integrates the Eulerian description of the fluid domain with the Lagrangian formulation of the moving mesh using curvature correction [32, 33]. The Newton–Raphson method was used to solve the nodal matrices [34, 35]. A first-order Euler backward implicit time integration method was used for the time marching.

### 2.4. Examined Parameters

The simulations examined several hemodynamic and biomechanical parameters in order to address the effect of the patch or primary suture in the carotid bifurcation, including effective stress, flow patterns, time-averaged WSS (TAWSS), and OSI. These parameters are known as critical factors in artery occlusion and thrombosis [36].

Wall stresses of the arterial tissue and artificial graft were calculated as the product of blood viscosity and the local velocity gradient in the direction of local surface normal (**n**):

(5) $$\begin{array}{c} \tau_{\text{w}}=\mu \frac{\partial u}{\partial n}. \end{array}$$

TAWSS and OSI were calculated according to Equations (6) and (7):

(6) $$\begin{array}{c} \text{TAWSS}=\frac{1}{T}\int_{0}^{T}\left|\tau_{\text{w}}\right|\;\;dt, \end{array}$$

(7) $$\begin{array}{c} \text{OSI}=0.5\left(1-\frac{\int_{0}^{T}\tau_{\text{w}}\;\;dt}{\int_{0}^{T}\left|\tau_{\text{w}}\right|\;\;dt}\right), \end{array}$$

where **τ**_w_ represents the instantaneous WSS vector and *T* represents the period of the cardiac cycle.

## 3. Results

### 3.1. Structural Results

**Figure 6:**
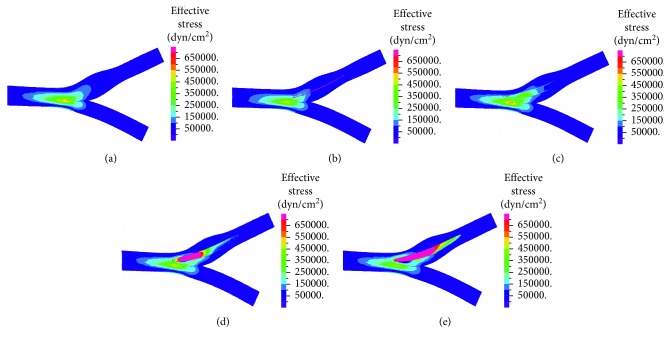
Effective stresses—top view in healthy artery (a), suture (b), high flexibility patch (c), medium flexibility patch (d), and low flexibility patch (e).

**Figure 7:**
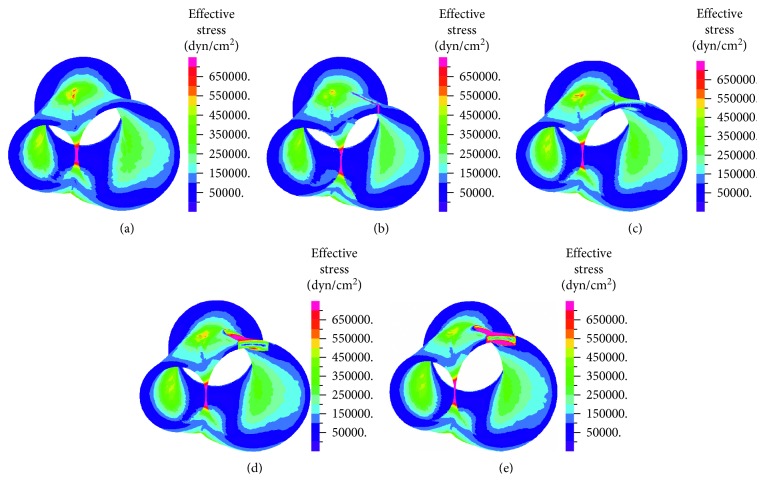
Effective stresses—cut view in healthy artery (a), suture (b), high flexibility patch (c), medium flexibility patch (d), and low flexibility patch (e).

Effective stresses at the wall were calculated at each point according to von Mises criteria. In all cases, elevated effective stresses were found at the bifurcation origin and bifurcation junction (Figures 6 and 7). In the suture and patches with medium and low flexibility cases, there were higher effective stresses (with values above 70 kPA) along the sutures. For the case with high flexibility patch, stress distribution resembled the values of healthy case.

## 4. Results of Fluid

**Figure 8:**
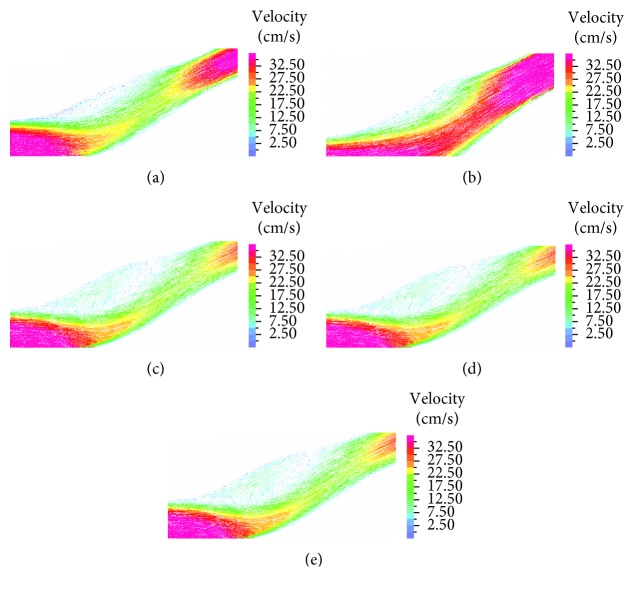
Velocity vectors—magnified view in healthy artery (a), suture (b), high flexibility patch (c), medium flexibility patch (d), and low flexibility patch (e).

Figure 8 presents midplane velocity vectors in a magnified view of the bulb for the five models after peak flow at the time *t* = 0.385 sec. In all five cases, a recirculation zone appears in the outer side of the bifurcation bulb. In the healthy case, the recirculation zone is small and most of the flow is unidirectional. In the model of the primary suture, the smaller diameter leads to a sustained velocity stream leaving room to a narrow vortex with less recirculation. In the patch model, although the diameter bulb is wide and the flow has plenty of room, the larger diameter promotes a large vortex that takes over almost the whole bulb space, which interferes with the axial flow stream.

**Figure 9:**
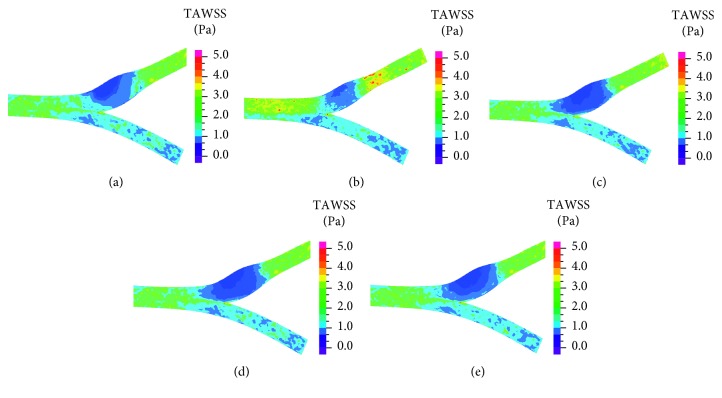
Time-averaged WSS in healthy artery (a), suture (b), high flexibility patch (c), medium flexibility patch (d), and low flexibility patch (e).

**Figure 10:**
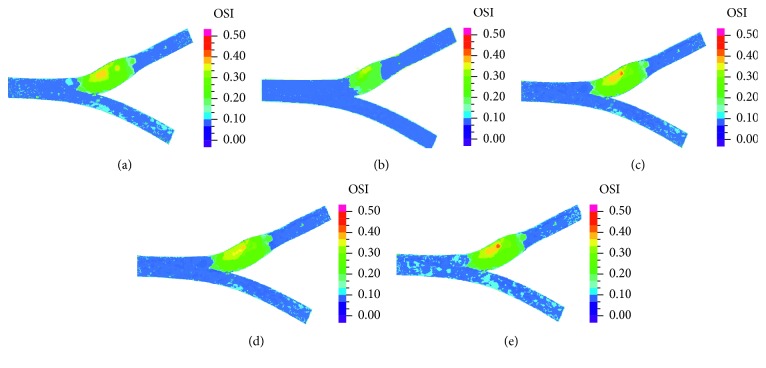
OSI in healthy artery (a), suture (b), high flexibility patch (c), medium flexibility patch (d), and low flexibility patch (e).

Figure 9 presents time-averaged wall shear stress (TAWSS), and Figure 10 presents the oscillatory shear index (OSI) distribution in the five models. Lower TAWSS and higher OSI values were present mostly in the bulb area, and low values of TAWSS and high values of OSI were rarely found in the primary suture case (in respect with the other cases) because of the smaller diameter in the bulb area.

## 5. Discussion

High-concentration stresses are found in the suture and low-flexibility patch cases (Figures 6 and 7). The stress concentrations are a result of the discontinuity of the material property of the patch and suture from the arterial wall. High-effective stress values are also found at the bifurcation junction. The values (in the range of 0.5–70 kPa) are in agreement with values reported by Kamenskiy et al. [22]. Concentration of stresses on the artificial graft might imply a higher risk of rupture. High stresses at the arterial wall might lead to atherosclerosis and neointima growth [37–39]. When there is stress concentration over a larger area (like in the low flexibility patch), the chances of developing restenosis are larger. The high flexibility patch resulted in lower stresses (>35 kPa), both at the artificial graft and the arterial wall. Therefore, in specific cases, when it is decided to prefer a patch over a suture in patients with relatively small arteries, high flexibility patch (such as vein or bovine patch) should result in lower risk for stress-induced restenosis because its properties resemble those of the artery. However, they have a larger risk of forming an aneurysm. Ultimately, the primary suture exhibits effective stress distribution in the artery similar to the healthy artery, indicating that, from our study, the primary suture has less potential effect on the arterial tissue.

From examination of flow patterns in the different models (Figure 8), we conclude that some vortical flow patterns are found in all cases but in different sizes. In a primary suture model, the flow in the bulb is mostly unidirectional and only a negligible vortex is found. In the patch and in the healthy models, the large diameter bulb promotes a large vortex that dominants the flow. According to Gimbrone et al. [40], vortices and disruptions in the flow are factors that cause a decrease in endothelial function and eventually might increase risk for stenosis or other vascular diseases. In addition, these vortices might disturb the flow and lead to thrombus formation and thus increase the risk for stroke.

The results of TAWSS (Figure 9) and OSI (Figure 10) reveal that WSS values in the bulb region are similar to the healthy case and the patches cases, while in the suture case, the values are higher. It can be concluded that bulb diameter is the cause for variations in WSS. In regions with larger diameter, the velocities are smaller, and therefore, WSS are smaller and OSI are higher. Low WSS and high OSI are correlated with plaque deposition, artery occlusion, and endothelium dysfunction [41]. Therefore, from our study, the suture case which has higher WSS and lower OSI is also preferred by this parameter. These results agree with Domanin et al. [26] that showed higher values of OSI and RRT in patch graft vs. direct suture cases in patient-specific simulations, and with Kamenskiy et al. [22] that showed that artery with a narrow patch showed significant improvement in hemodynamics in comparison with wider patches.

The influence of fluid shear forces on structure dynamics is relatively small. WSS is negligible (<70 dyn/cm^2^) compared to the effected stresses due to hydrostatic pressure (<70,000 dyn/cm^2^). Therefore, when a simplified simulation is needed for estimation of local effective stresses, it may be valid to consider the structural domain separately from the fluid domain.

The study assumes a relatively simplified model of a specific anatomy, with linearly elastic material and Newtonian fluid, and does not consider possible physiological or anatomic variations between healthy and post-CEA [29]. The actual values are highly geometry-dependent and therefore, patient-specific. Moreover, in reality, during the CEA procedure, a part of the media layer is removed, with only adventitia layer remaining. This might further increase the effective stress in the bifurcation wall in relation with the healthy case and increase the risk for rapture or aneurysm. In addition, the results and conclusions discuss only the mechanical aspects and did not take the clinical aspect as argument.

Another limitation relates to the time and space discretization parameters. Note that a time-step interval of 0.035 sec is relatively elevated, in respect of systolic interval (of 0.4 sec), and may limit capturing the systolic fluid dynamics. To minimize this limitation, an automatic-time-stepping procedure was used to subdivide the load-step increment when necessary. According to our mesh and time discretization refinement studies (Appendix), the discretization errors in the calculation of WSS are up to 10%.

However, the model results are in agreement with clinical and previous studies [25, 26, 42], and the comparative results between the different models are clear and distinct. Most guidelines suggest to prefer primary suture for narrow ICA (>4 mm), and the current model assumes ICA with 5 mm, showing preferred performance with primary patch. This study may delineate the dominant parameters affecting the combined hemodynamic and biomechanics of the patches versus suture approach. Thus, its results may shed a light on the controversy between physicians regarding the preferred approach and explain the reason for the nonsignificant advantage of the patch procedure in CEA.

## 6. Conclusion

In this manuscript, we examined the biomechanical aspects of patch angioplasty in comparison with primary suture. The examined parameters included elevated stress, in addition to the previously examined hemodynamic (WSS) parameters. Based on our results of elevated stress and OSI values and low TAWSS values, primary suture has shown better performance in our study than patch, and the high flexibility patch has shown better performance compared to lower flexibility patch.

## Figures and Tables

**Table 1:** Cases studied.

Case#1	A healthy carotid bifurcation: a time-dependent simulation of the coupled domains

Case#2	A narrowed postoperation carotid bifurcation with a suture: a time-dependent simulation of the coupled domains

Case#3	A widened carotid bifurcation with a *high* flexibility patch: a time-dependent simulation of the coupled domains

Case#4	A widened carotid bifurcation with a *medium* flexibility patch: a time-dependent simulation of the coupled domains

Case#5	A widened carotid bifurcation with a *low* flexibility patch: a time-dependent simulation of the coupled domains

**Table 2:** Geometric dimensions of the bifurcation models.

Dimension	Labels in Figure 1	Value (mm)		
		Healthy	Suture	Patch
CCA internal diameter	*a*	6.6		
Max. bulb internal diameter	*b*	7.8	7.0	8.4
Max. bulb location	*c*	7.7		
Bulb length	*d*	16.0		
ICE internal diameter	*e*	5.0		
ECA internal diameter	*f*	4.7		
Wall thickness	*g*	0.7		
Patch width	*h*			8.0
Patch length	*i*			18.0
Patch thickness		0.7	0.7	0.7
ICA bifurcation	*θ*_1_	25°		
ECA bifurcation	*θ*_2_	25°		

**Table 3:** Material properties.

*ν*	*E* (^*∗*^10^6^ dyn/cm^2^)	*ρ* (g/cm^3^)	*µ* (dyn^*∗*^s/cm^2^)	
		1.05	3.5 *∗* 10^−2^	Blood
0.499	5	1.05		Artery
0.499	10	1.05		High flexibility patch
0.499	200	1.05		Medium flexibility patch
0.499	800	1.05		Low flexibility patch
0.37	2000	1.35		Suture

**Table 4:** Resulted values with different time steps (error in % is indicated in parentheses).

Time step (sec)	0.035	0.01
Max. velocity magnitude (cm/s)	58.5 (1.8%)	59.5
Max. shear stress (dyn/cm^2^)	122.1 (1.7%)	124.2
Max. effective stress (dyn/cm^2^)	2.12 *×* 10^7^(1.8%)	2.15 *×* 10^7^

**Table 5:** Maximum values for different mesh resolutions (error in % is indicated in parentheses).

No. of elements	51,544	100,268	484,401
Max. velocity magnitude (cm/s)	57.5 (5.7%)	58.5 (2.7%)	60.1
Max. shear stress (dyn/cm^2^)	94.7 (28.1%)	122.1 (3.5%)	126.4
Max. effective stress (dyn/cm^2^)	1.64 *×* 10^7^ (28.9%)	2.12 *×* 10^7^(8.5%)	2.21 *×* 10^7^

## Data Availability

The data used to support the findings of this study are included within the article and appendix. Raw data files of the models and simulations can be released upon application to the corresponding author (iditav@ariel.ac.il).

## Appendix

### A. Model Validation

#### A.1. Time-Step Independence Tests

Mesh and time-step independence tests were conducted to validate the numerical model. To evaluate the optimal time-step size for the transient simulations, FSI simulations of the healthy case with the mesh of 100,000 elements were performed using 28 steps of 0.035 sec and 98 steps of 0.01 sec. The resulted maximal values in the domains for the two cases are detailed in Table 4.

To evaluate the discretization error, we calculated the relative difference (ERR) between the maximal value in the course and the fine time resolution, as follows:

(A.1) $$\begin{array}{c} {\text{ERR}}_{i}\left(\%\right)=\left(\frac{{\left(X\right)}_{\text{fine}}-{\left(X\right)}_{\text{course}}}{{\left(X\right)}_{\text{fine}}}\right)\;*\;100\left(\%\right), \end{array}$$

where *X* is the maximal value in the domain (velocity and shear stress in the fluid domain and effective stress in the structural domain). The resulted errors are detailed in the table. The results show that the time step of *dt* = 0.035 sec is sufficient, with ERR < 2% in the three critical parameters. Therefore, for the transient analyses in this study, 28 steps were set per cycle.

Note that only maximum values were examined (in space and time), thus instantaneous differences at a given instance (e.g., systole) were not examined. Note that this time-step interval is relatively elevated, in respect of systolic interval (of 0.4 sec), and may limit capturing the systolic fluid dynamics. To minimize this limitation, an automatic-time-stepping procedure was used to subdivide the load-step increment when necessary.

#### A.2. Mesh Independence Tests

To evaluate the optimal mesh resolution, three models of the healthy base case with different mesh resolutions were built (with 50,000–500,000 elements). The models were simulated during a period of one cardiac cycle, each with 28 time steps. The resulted maximal values in the domains of each model mesh are listed in Table 5, together with their error relative to the finest mesh (Equation (A.1)). Based on these results, mesh resolutions of 100,000 elements were found suitable for our model with ERR ≤ 10% of the finest mesh.
